# Allosteric Communication of the Dimerization and the Catalytic Domain in Photoreceptor Guanylate Cyclase

**DOI:** 10.1021/acs.biochem.4c00170

**Published:** 2024-08-23

**Authors:** Manisha Kumari Shahu, Fabian Schuhmann, Siu Ying Wong, Ilia A. Solov’yov, Karl-Wilhelm Koch

**Affiliations:** † Department of Neuroscience, Carl von Ossietzky Universität Oldenburg, Carl-von-Ossietzky-Str. 9-11, 26129 Oldenburg ,Germany; ‡ Niels Bohr International Academy, Niels Bohr Institute, University of Copenhagen, Blegdamsvej 17, 2100 Copenhagen, Denmark; § Institute of Physics, Carl von Ossietzky Universität Oldenburg, Carl-von-Ossietzky-Str. 9-11, 26129 Oldenburg, Germany; ∥ Research Centre for Neurosensory Science, Carl von Ossietzky Universität Oldenburg, Carl-von-Ossietzky-Str. 9-11, 26129 Oldenburg ,Germany; ⊥ Center for Nanoscale Dynamics (CENAD), Institute of Physics, Carl von Ossietzky Universität Oldenburg, Ammerländer Heerstr. 114-118, 26129 Oldenburg, Germany

## Abstract

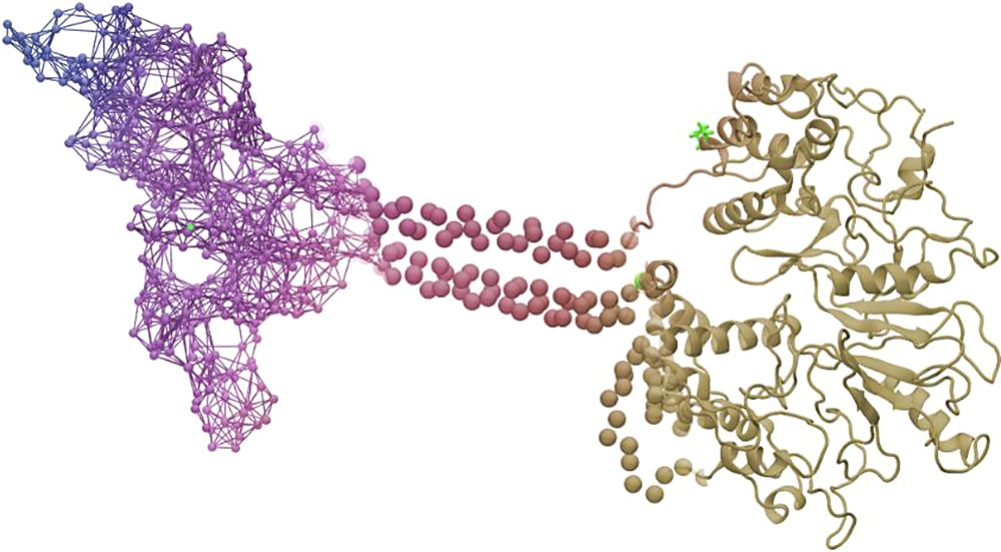

Phototransduction in vertebrate photoreceptor cells is controlled by Ca^2+^-dependent feedback loops involving the membrane-bound guanylate cyclase GC-E that synthesizes the second messenger guanosine-3′,5′-cyclic monophosphate. Intracellular Ca^2+^-sensor proteins named guanylate cyclase-activating proteins (GCAPs) regulate the activity of GC-E by switching from a Ca^2+^-bound inhibiting state to a Ca^2+^-free/Mg^2+^-bound activating state. The gene *GUCY2D* encodes for human GC-E, and mutations in *GUCY2D* are often associated with an imbalance of Ca^2+^ and cGMP homeostasis causing retinal disorders. Here, we investigate the Ca^2+^-dependent inhibition of the constitutively active GC-E mutant V902L. The inhibition is not mediated by GCAP variants but by Ca^2+^ replacing Mg^2+^ in the catalytic center. Distant from the cyclase catalytic domain is an α-helical domain containing a highly conserved helix-turn-helix motif. Mutating the critical amino acid position 804 from leucine to proline left the principal activation mechanism intact but resulted in a lower level of catalytic efficiency. Our experimental analysis of amino acid positions in two distant GC-E domains implied an allosteric communication pathway connecting the α-helical and the cyclase catalytic domains. A computational connectivity analysis unveiled critical differences between wildtype GC-E and the mutant V902L in the allosteric network of critical amino acid positions.

## Introduction

Inherited retinal dystrophies (IRDs) are widespread and affect millions of people worldwide. They are both phenotypically and genotypically heterogeneous diseases^1−3^ and include retinitis pigmentosa (RP), Leber congenital amaurosis (LCA), and cone-rod dystrophy (CRD). Causes of retinal dysfunction are often pathological changes in the function and operation of light-sensitive rod and/or cone photoreceptor cells. Photoreceptor cells mediate the sensory phototransduction process of the visual system by converting the absorbance of photons to electrical signals. Visual information is further transmitted via retinal neurons and is decoded by the visual processing center of the brain. Phototransduction in vertebrate rod and cone cells depends critically on the homeostasis of two second messengers, guanosine-3′,5′-cyclic monophosphate (cGMP) and Ca^2+^. The cytoplasmic concentrations of both messengers decrease after illumination and recover by deactivation and feedback processes.^4−7^ An imbalance of their cytoplasmic concentrations has detrimental effects and leads to visual dysfunction and even blindness in humans. According to a comprehensive list of data on RetNet (https://sph.uth.edu/retnet/), more than 300 genes are associated with IRDs, and over 140 disease-causing mutations described so far are found in the *GUCY2D* gene.^3^*GUCY2D* is the gene that encodes for photoreceptor guanylate cyclase GC-E (also known as ROS-GC1 or retGC1), a key enzyme in phototransduction that synthesizes the second messenger cGMP and returns the cell to its dark-adapted state in a Ca^2+^-dependent negative feedback loop.^5,7^

Cytoplasmic Ca^2+^-sensor proteins named guanylate cyclase-activating proteins (GCAPs) mediate this feedback on GC-E activity by activating GC-E at a low Ca^2+^ concentration [Ca^2+^] when GCAPs switch to a Mg^2+^-bound activating form. GC-E returns to the basal, low-activity state, when GCAPs are saturated with Ca^2+^.^8−10^ The functional state of the GC-E requires a homodimeric topology that consists of an extracellular (ECD), a transmembrane (TMD), and an intracellular domain (IcD). The IcD consists of a juxtamembrane (JMD), a kinase homology (KHD), a dimerization (DD), and a catalytic (CD) domain. Rehkamp et al.^11^ presented the first 3D structural data for the IcD of GC-E, combining cross-linking and mass spectrometry of a native GC-E preparation with computational modeling. Rehkamp et al.^11^ modified a previous division of the IcD by suggesting a novel domain organization formed of a KHD, an “α-helical domain” (αHD), and the cyclase catalytic domain (CCD). The αHD connects the KHD with the CD and contains a highly conserved helix-turn-helix motif at its N-terminal extension found in topologically related proteins.^12^ It also includes the formally assigned DD. Structural studies on related soluble guanylate cyclases point to an intrinsic flexibility in the hinge motif of the αHD leading to various conformations. For example, a conformational change from a 90° kinked helix-turn-helix motif (PDB: 6PAS representing the inactive state) to a straight helix motif (PDB: 6PAT representing the active state) triggers the activation of the soluble guanylate cyclase from *Manduca sexta*.^13^ This high degree of flexibility is also observed in the αHD of the GC-E^11^ and, therefore, is the focus of current research. The domain is critical for dimerization and is suggested as a GCAP binding interface or regulatory control module.^14,15^

Several amino acid positions are mutated in the GC-E of human patients suffering from autosomal dominant cone-rod dystrophy (adCRD), making this a “hot spot” region for retinal diseases.^3^ In addition, mutations in GC-E are spread over all domains, and functional studies addressing the effects of point mutations often indicate a drastic decrease in GC-E activity. However, exceptions as we described for the point mutation V902L in GC-E lead to a constitutively active form exhibiting high activity in the absence and presence of GCAP1 thereby exceeding the suggested physiological level of the cGMP synthesis rate.^16^ Patients carrying the point mutation V902L suffer from CRD,^16^ which is probably caused by the impaired ability of the mutant GC-E to return to the low-activity state during recovery of the photoresponse.

Kinetic analysis of enzymatic parameters and molecular dynamics (MD) simulations of the V902L mutant suggested a swinging movement of the dimerization domain in the V902L mutant as the critical switch to transit to the GC-E active state.^17^ This indicates that a point mutation in the CD, such as V902L, triggers a movement upstream in the GC-E structure, indicating robust connectivity between the αHD and the CD.

These observations show that the precise responses of photoreceptors require tightly controlled conformational transitions in GC-E. Inspired by kinetic and structural studies on soluble guanylate cyclases,^13,18^ we investigated this transition further using a combination of experimental enzymatic assays and MD simulation. We investigated a double mutant with critical mutations in the helix-turn-helix motif (L804P) and the CD (V902L) to test the suggested allosteric communication between the αHD and the CD. We investigated how the GCAP-independent Ca^2+^ sensitivity of the mutant V902L can be understood and how this relates to the active or inactive state in the catalytic center. Finally, we performed a computational connectivity analysis to construct an amino acid interaction network operating in conformational transitions.

## Results and Discussion

### Effects of Ca^2+^ on the Catalytic Center

**Figure 1:**
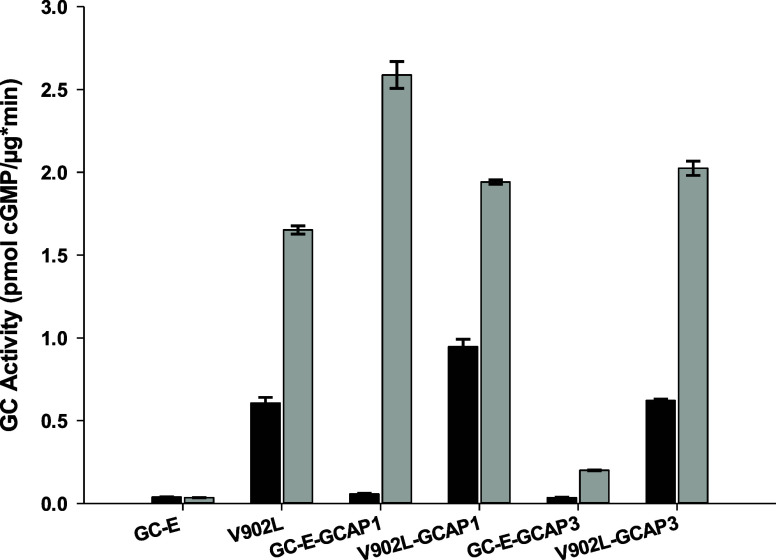
Activity profiles of GC-E variants. WT and mutant V902L were activated with GCAP1 or GCAP3 at high (33 μM, black bars) or low (<10 nM, gray bars) free [Ca^2+^]. The *x*-fold activation of V902L without GCAPs is 2.7, in the presence of GCAP1, 2.0, and in the presence of GCAP3, it is 3.3. The *x*-fold activation of GC-E WT is 45.5-fold with GCAP1 and 5.6-fold with GCAP3. Error bars are s.d. (one example of technical triplicates out of three to four biological replicates; results of other biological replicates are shown in the Supporting Information, Figures S1–S3).

Our previous work indicated an allosteric communication pathway between the helix-turn-helix region in the αHD and the CD.^17^ The point mutant V902L appears as a useful tool for investigating the properties of this allosteric effect further. First, we analyzed whether the reduced efficiency of GCAP1 activating GC-E was observed with the other GCAP variant, GCAP3. Figure 1 shows the activity profiles of WT and mutant V902L in the presence of either GCAP1 or GCAP3 at high and low [Ca^2+^]. The mutant V902L exhibited a high activity at high and low [Ca^2+^] in the absence of any GCAP variant exceeding the basal activity 6- to 16-fold (compare columns WT and V902L in Figure 1). An addition of GCAP1 has only a modest, however significant, effect on the activity of V902L in the presence and absence of Ca^2+^ (Figure 1, V902L vs V902L-GCAP1). These results confirm our previous conclusion that the mutant V902L is a constitutively active GC-E.^16^ Interestingly, GCAP3 activated GC-E in a Ca^2+^-dependent manner, but to a lesser degree than GCAP1, whereas mutant V902L was almost unaffected by GCAP3 (Figure 1). GC activities of V902L in the presence and absence of GCAP3 at high [Ca^2+^] were almost the same (Figure 1). At low [Ca^2+^], GCAP3 caused slightly higher activity of V902L (Figure 1).

**Figure 2:**
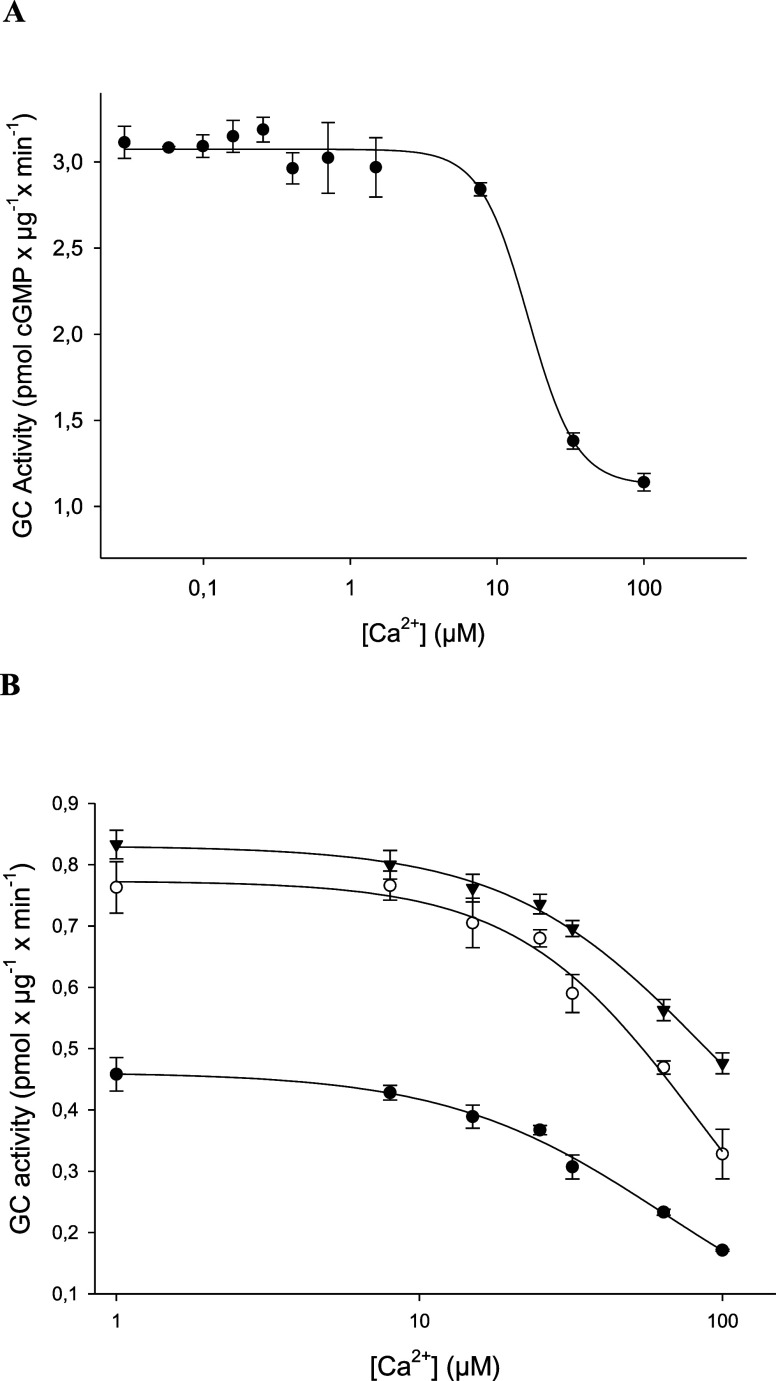
Ca^2+^-dependent inhibition of the V902L mutant. (A) Incubation of V902L was performed in the absence of GCAP1 or GCAP3. Free [Ca^2+^] was varied, as indicated, and half-maximal inhibition was at 16.3 μM free [Ca^2+^] (error bars are s.d., one example of technical triplicates out of three biological replicates, see also Figure S4). (B) Increasing concentrations of free Mg^2+^ reverse the inhibitory effect of Ca^2+^. Incubation was performed as in (A). The Mg^2+^ concentration was 2 mM (●, filled circles), 3.5 mM (**○**, open circles), and 5 mM (▼, filled triangles). We performed nonlinear regression fitting using a sigmoidal Hill model provided by the SigmaPlot 13.0 software (three-parameter model for data with 2 and 3.5 mM Mg^2+^; four-parameter model for 5 mM Mg^2+^). Error bars are standard deviation (s.d.) (technical triplicates). All curve fittings passed the normality test (Shapiro–Wilk) and the constant variance test.

The activity profile of V902L in the absence of GCAPs (Figure 1) showed that the activity of mutant V902L is Ca^2+^-dependent, although no Ca^2+^-sensor protein is present to mediate this effect. We measured the V902L activity with increasing free [Ca^2+^] and observed a nearly constant activity of V902L up to 10 μM free [Ca^2+^] (Figure 2A). Above 10 μM free [Ca^2+^], the activity decreased and reached a plateau of lower activity at 100 μM free [Ca^2+^] slightly below the activity observed at a free [Ca^2+^] of 33 μM as shown in Figure 1. Half-maximal inhibition (IC_50_) was observed between 16 and 19 μM free [Ca^2+^] employing a sigmoidal four-parameter Hill model provided by the SigmaPlot 13.0 software, Systat Software, Inc., San Jose, CA, 2014 (two independent data sets in triplicates, see example in Figure 2A). The constitutive activity of the V902L mutant allowed us to dissect the Ca^2+^ dependency of GC-E mediated by GCAPs from a direct Ca^2+^ effect on the catalytic mechanism. Serfass et al.^18^ previously analyzed a similar Ca^2+^-dependent inhibition for the catalytic properties of soluble guanylate cyclase from bovine lung. The authors interpreted their finding as a two-metal ion catalytic mechanism involving two Mg^2+^ ions similar to the catalytic mechanism observed in adenylate cyclases.^19−21^ One Mg^2+^ forms with GTP the substrate complex and one Mg^2+^ (in excess of the substrate) is primarily bound to the cyclase in the catalytic center. Such a mechanism implies that Ca^2+^ bound to the cyclase can be removed by increasing the free Mg^2+^ in excess of the Mg^2+^-GTP substrate. We performed an experiment to reverse the inhibition of Ca^2+^ at three different Mg^2+^ concentrations (Figure 2B) and observed two effects: GC activity increased reaching a saturating profile at an excess of 5 mM Mg^2+^ and 100 μM Ca^2+^ were not sufficient for an inhibitory effect as seen in Figure 2A. Fitting data points in Figure 2B revealed IC_50_ at Ca^2+^ concentrations of 64, 82, and 91 μM for Mg^2+^ concentrations of 2, 3.5, and 5 mM, respectively. Differences were statistically significant for curves with 2 and 3.5 mM Mg^2+^ (*p* < 0.01; *t* test) but not when comparing curves for 3.5 and 5 mM Mg^2+^ (n.s.).

### Critical Mutation in the Helix-Turn-Helix Motif Has an Impact on the Catalytic Center

**Figure 3:**
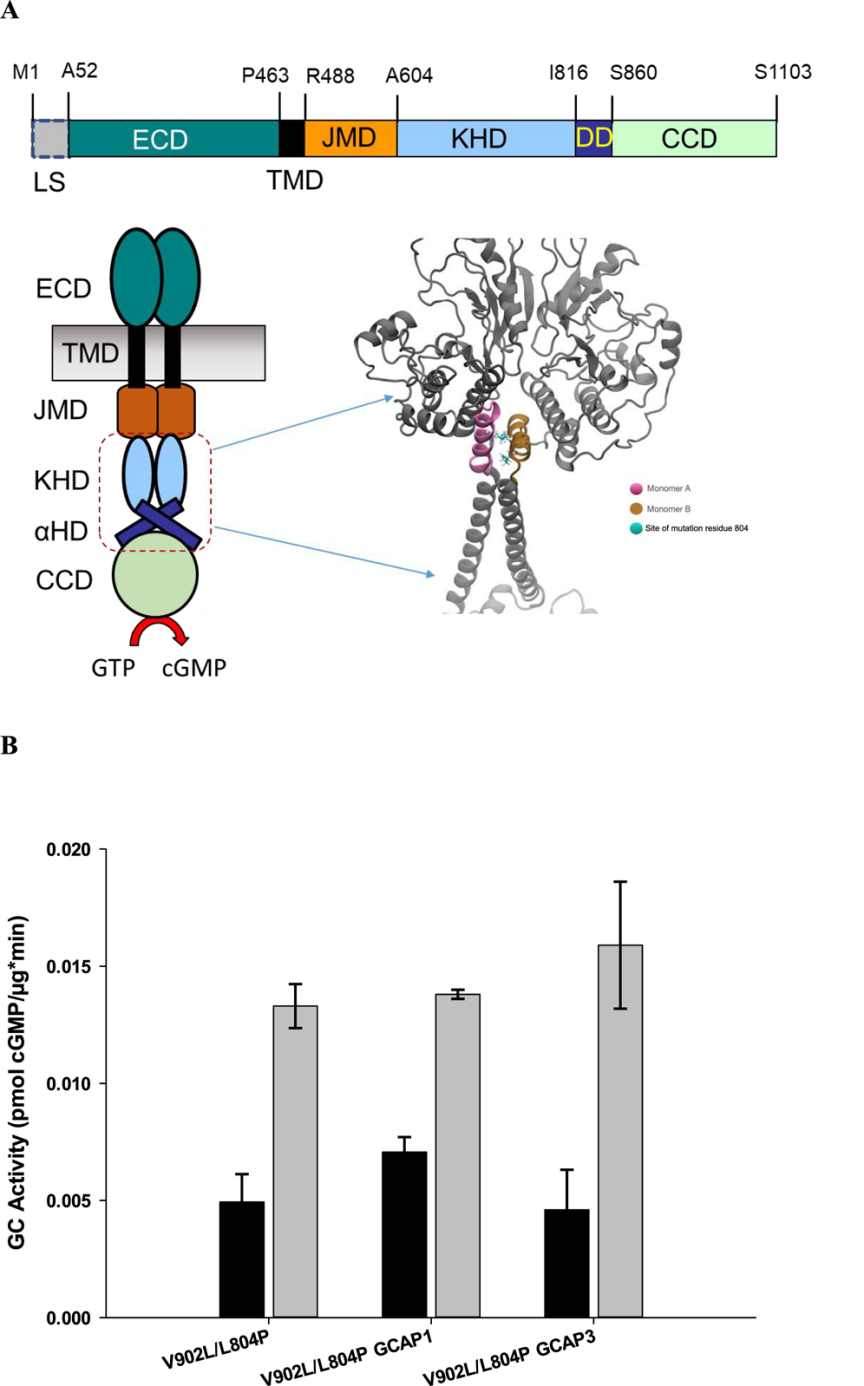
Activity profiles of the GC-E variants. (A) Upper part: general topology of a GC-E monomer showing domain locations (LS, leader sequence; ECD, extracellular domain; TMD, transmembrane domain; JMD, juxtamembrane domain; KHD, kinase homology domain; DD, dimerization domain; CCD, cyclase catalytic domain). Amino acid positions indicate the start of the respective domain, and S1103 indicates the last amino acid of the primary sequence. Lower part: location of the point mutation L804P in the helix-turn-helix motif. (B) Activity of the double mutant V902L/L804P in the absence and presence of GCAP1 at high (33 μM, black bars) or low (<10 nM, gray bars) free [Ca^2+^]. The x-fold activation of the double mutant without GCAPs is 2.7, in the presence of GCAP1, 2.0, and in the presence of GCAP3, it is 3.4. Comparing activities of the Ca^2+^-bound states yielded no significant differences for all combinations (V902L/L804P vs V902L/L804P + GCAP1, V902L/L804P + GCAP1 vs V902L/L804P + GCAP3, and V902L/L804P vs V902L/L804P + GCAP3). Error bars are s.d. (one example of technical triplicates out of three to four biological replicates; results of other biological replicates are shown in the Supporting Information, Figures S5–S7).

Switching GC-E back to the basal activity state is a decisive step in phototransduction. Our previous simulations pointed to a conformational change in the αHD required for GC-E to transit between low- and high-activity states. Since we located the structural trigger of the switch in the helix-turn-helix motif, we reasoned that changing or breaking a critical α-helix might impact the catalytic center. This suggestion is further supported by previous findings that locate a crucial regulatory control module in or near the dimerization domain.^14,15^ Therefore, to evince our hypothesis, we substituted leucine at position 804 with proline in the V902L mutant, creating the double mutant L804P/V902L (LP-VL) (Figure 3A).

Subsequent testing of GC activities showed two main effects: a general decrease in activity in the absence and presence of GCAPs (Figure 3B). The V902L/L804P mutant has a reduced basal activity in comparison to WT GC-E. The activity is 8-fold lower in the presence of Ca^2+^ and 3-fold lower in the presence of EGTA (comparison of Figure 1, data shown for *GC-E,* with Figure 3, data shown for V902L/L804P). Expression of V902L and the double mutant was identical as tested by Western blotting and immunohistochemistry of transfected HEK 293 cells (supplements, Figures S8 and S9). However, biological replicates differ in expression yield of GC-E variants (see the Supporting Information). Second, we compared the x-fold activation (activity at low [Ca^2+^] divided by activity at high [Ca^2+^]) of the mutant V902L with the double mutant and the WT (see the legend of Figure 3B) yielding an *x*-fold activation for both mutants between 2- and 4-fold indicating that the principal activation mechanism remained intact but at a lower level of catalytic efficiency. Comparing all Ca^2+^-bound states with each other (double mutant V902L/L804P with GCAP1 or GCAP3) yielded no significant differences (Figure 3B). The same was observed for the Ca^2+^-free states. Compared to WT GC-E (*x*-fold activation is more than 45-fold, Figure 1), we observed no recovery of the Ca^2+^-sensitive GCAP effect in the double mutant but a substantial effect on the catalytic performance. Our results supported our hypothesis of a conformational pathway connection between the αHD and the CD.

### Connectivity Analysis

Our experimental analysis of critical amino acid positions in two distant GC-E domains implied a communication pathway connecting the αHD and the CD. To achieve a broader view of the allosteric regulatory impact, we carried out a computational connectivity analysis, as described in the Methods section.

The connectivity analysis utilizes two approaches that probe separate attributes describing connectivity within a network derived from the locations of amino acid residues during MD simulations. The *reach method*(22) values amino acids that reach the most other amino acids along the network edges as highly influential. On the other hand, the *betweenness method*(23) prefers amino acid residues, which are located at important network intersections throughout the protein structure.

**Figure 4:**
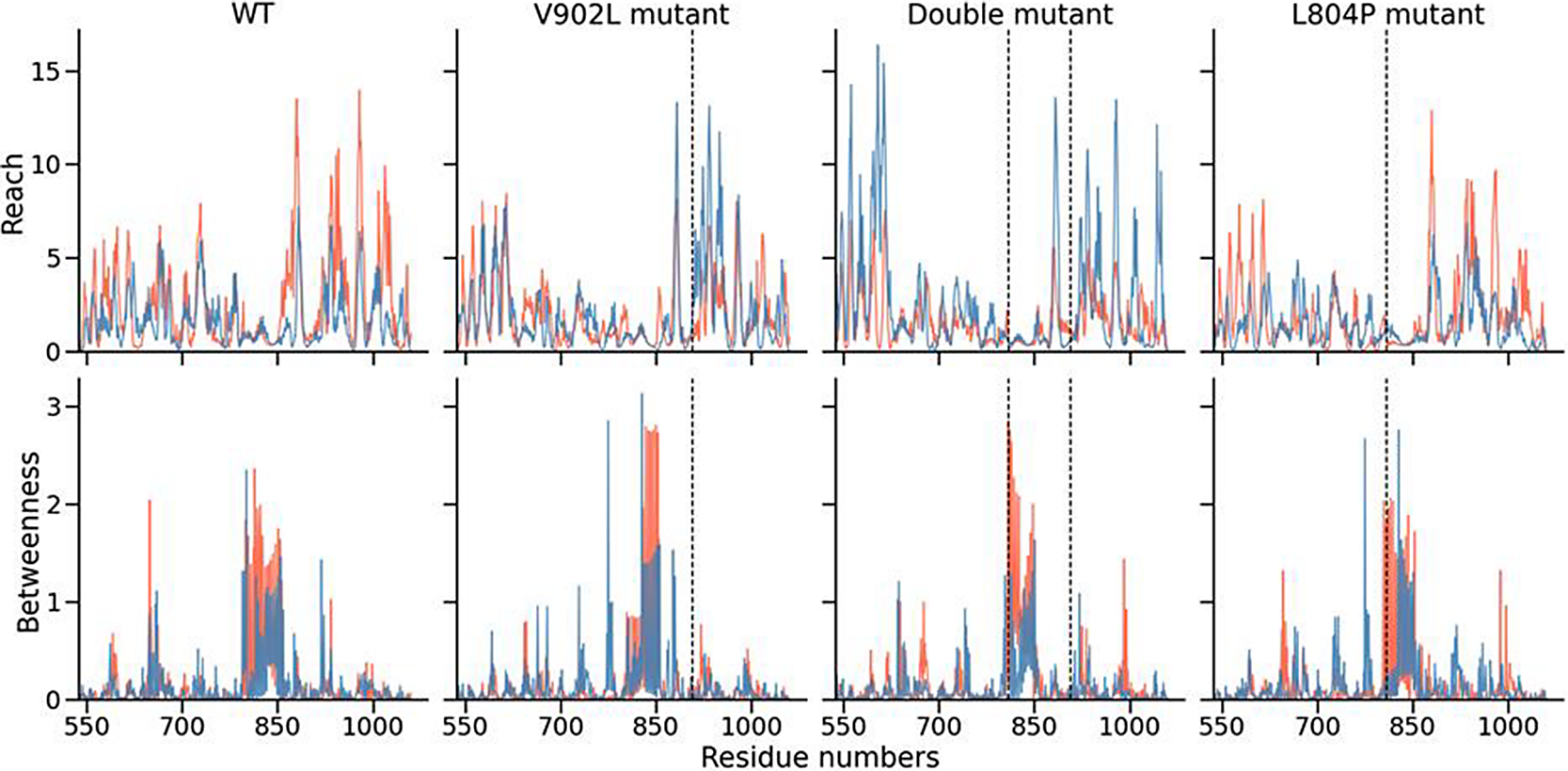
*Reach* and *betweenness* of the monomers for all residues. The graphs show the *reach* (first row) and *betweenness* (second row) of the two monomers. The first monomer is colored blue, the second red. The vertical dashed lines indicate mutated residues at positions 902 and 804. Noticeably, considering the introduction of further mutations (WT, V902L, and double mutant), the reach of the residues within the homology kinase domain increases with the introduction of more mutations, while the importance of the different monomers switches for the catalytic domain. Contrary to the *reach*, the *betweenness* picks up on the αHD. Additionally, the *betweenness* is greater in the second monomer after introduction of the single mutant. The effect is more diversified again in the double mutant, however, still leaning toward the red mutant. Interestingly, almost isolated signals can also be found in the two other domains. Furthermore, the second single mutant L804P remains similar to the wildtype protein, except for a slight shift to a single monomer in the betweenness in the αHD, which can also be observed in the double mutant.

The graphs in Figure 4 show the *reach* (first row) and *betweenness* (second row) for the individual amino acid residues. The results align with the assumption that the residues with the highest *betweenness* are located in the αHD, which connects the homology kinase and catalytic domains, as all shortest paths from one domain to the other have to pass through these residues. The residues with the highest *reach* are located in the catalytic domain for the wildtype and the V902L mutant. For the double mutant, however, residues in the homology kinase domain have a similar *reach* to those in the catalytic domain. In particular, the residues with a high gap seem to shift toward a single monomer, while concentrated in the αHD for the single mutant. While the effect is still visible in the double mutant, it is more diversified and reduced, particularly for residues in the middle of the αHD. The L804P mutant is remarkably similar to the wildtype with a slight exception in the differences in the αHD. Here, the high-moiety residues conglomerate in a single monomer, which can also be seen in the double mutant.

**Figure 5:**
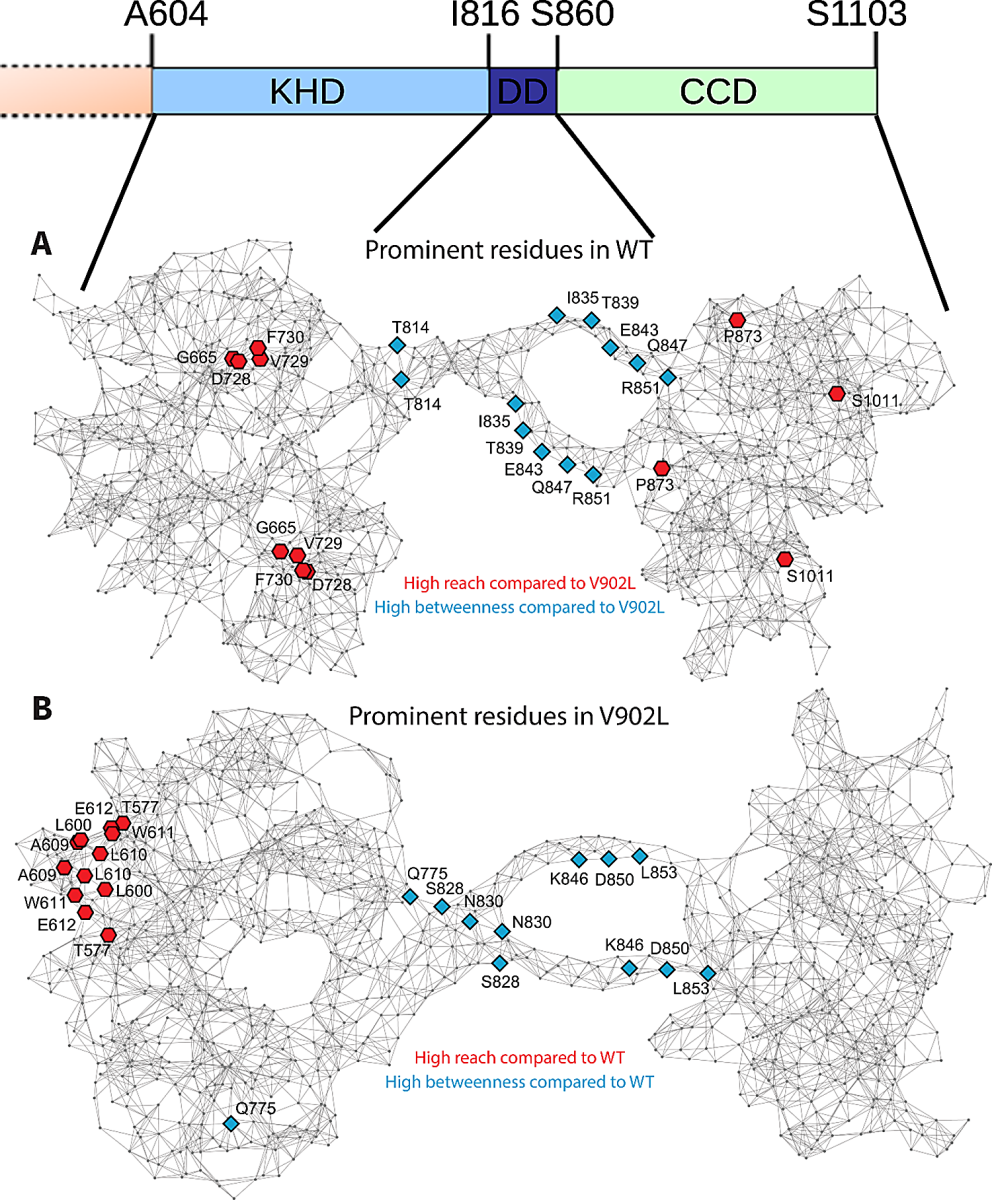
Graphical network representations of the WT (A) and V902L (B) proteins, plotted in gray using the spring embedding method in Wolfram Mathematica (https://www.wolfram.com/mathematica). The six residues with the highest *reach* (red hexagons) and *betweenness* (blue diamonds) compared to the other protein version (WT and V902L mutant, respectively) are highlighted and labeled in each graph. Corresponding domains and amino acid positions are indicated on top.

With these *betweenness* graphs (Figure 4) and averaging of the two monomers, potential residues of importance can be singled out. The analysis was performed for the WT and the V902L mutant. The six residues with the most significant *reach* and *betweenness* (relative to each other) are shown in Figure 5. The *high-betweenness* residues are located in the double helix of the αHD and do not differ significantly between the WT and the V902L mutant. The *reach*, however, paints a different picture. The residues with the most significant *reach* change from the WT to the V902L mutant. In the mutant, the highest *reach* residues are located at the N-terminal part of the homology kinase domain, almost at the juxtamembrane domain (not displayed and modeled). In the WT, the highest *reach* residues are even split across the homology kinase and catalytic domains. Here, the residues are also closer to the αHD.

### Functional Interpretation of the Connectivity Results

Marino and Dell’Orco^24^ performed a structural network analysis of GCAP1 and described an allosteric information transfer from different cation binding states to amino acid residues in the GC-E target interface.^24^ The same authors extended the analysis to related neuronal Ca^2+^-sensor proteins and uncovered conserved amino acid positions in three related Ca^2+^-sensor proteins that point to an evolutionary conservation of molecular communication pathways.^25^

Our analysis presented here focuses on the GCAP1 target GC-E and the amino acid network involved in the conformational transition process. For example, position S1011 in the CD is next to E1010, which forms a complex with Mg^2+^ and the pyrophosphate part of GTP.^26^ The loss of *high reach* in V902L (Figure 5B) might be the reason for the decrease or even loss of the allosteric regulation by GCAPs as seen in the functional properties of the V902L mutant (refs (16) and (17); this study). The remaining Ca^2+^-sensitive control is located in the binding site of GTP in the catalytic center and might significantly impact the V902L mutant under certain conditions. For example, constitutive activation of mutant V902L would create a high level of cytoplasmic cGMP in the photoreceptor cell, causing a higher influx of Ca^2+^ than average. However, an excessive local increase in concentrations of Ca^2+^ would then inhibit V902L by the direct binding of Ca^2+^, bringing the cGMP synthesis back to low basal rates. This scenario would still differ from a healthy state since it has shifted Ca^2+^–cGMP homeostasis.

Two amino acid positions with a *high reach* in the WT compared to those in the V902L mutant (Figure 5) are mutated in patients suffering from retinal diseases. One is the point mutation D728N in the KHD, causing autosomal recessive LCA.^27,28^ The second is P873R, located in the CD and found in patients suffering from CRD.^16^ A functional analysis of the latter showed a complete loss of GC-E activity, indicating a severe impact on photoreceptor physiology.^16^ Functional analysis of the D728N mutant is lacking so far. Still, mutations in the KHD often correlate with either impaired basal GC activity or partial loss of activity regulation by GCAPs (for a summary, see ref (3)). Thus, our connectivity analysis highlighted amino acid positions that are critical for the allosteric control of GC-E activity, and some of the positions presented in Figure 5 might carry point mutations that correlate with retinal diseases but are not detected so far.

## Conclusions

Switching of photoreceptor GC-E from the inactive state to the active state and *vice versa* is essential for second messenger homeostasis in rod and cone cells. Here, we describe an allosteric communication pathway linking two distant domains of GC-E and identify a network of amino acid positions that seem critical for enzyme activity control. Our connectivity analysis identified point mutations at some of these positions found in patients suffering from retinal dysfunction. Thus, the analysis might be able to predict positions that could cause retinal diseases when mutated.

## Methods

### Generation of the Double Mutant by Site-Directed Mutagenesis

The pIRES2-eGFP vector containing the GC-E mutant V902L sequence was used for amino acid substitution to generate the double mutant. Proline was introduced at position 804 to substitute for leucine (L804P) by site-directed mutagenesis, which was achieved by the polymerase chain reaction (PCR) using a KOD (Hot Start DNA Polymerase, Novagen) enzyme. The PCR amplification was followed according to the manufacturer’s protocol using a forward primer 5′-ATCAACAAGGGCCGGAAGACGAACATCATT-3′ and a reverse primer 5′-GTTCTTGAACGGGTCGAAGGTGTGGTCCAT-3′. The obtained clones were verified by full-length sequencing for the substitution of L804P.

### Heterologous Expression of the V902L Mutant and the L804P/V902L Double Mutant

The HEK293 cell line was transiently transfected with cDNA of GC-E mutants V902L and L804P/V902L using polyethylenimine (PEI) at 60–70% confluency in 100 mm plates. Respective 8 μg of DNA was mixed with 32 μg of PEI in DMEM without supplements and incubated at room temperature for 20 min. The sample mix was then added to the respective cell plate and incubated in the incubator at 37 °C with 5% CO_2_. After transfection and 72–96 h incubation, the cells were harvested by centrifugation at 500 × *g* for 5 min. The cell pellets were washed with PBS and centrifuged at 12,000 × *g* for 5 min. The pellets were frozen and stored at −80 °C until further use.

### Expression and Purification of GCAP1

Human myristoylated GCAP1 was expressed in *Escherichia coli* and purified to homogeneity by anion exchange chromatography and size exclusion chromatography as previously described and reported.^29^ Myristoylation of GCAP1 during bacterial expression was accomplished by cotransforming *Escherichia coli* cells with N-myristoyl-transferase from yeast and supplementation with myristic acid, as reported previously.^29^

### Expression and Purification of GCAP3

Human myristoylated GCAP3 was expressed in *Escherichia coli* and purified to homogeneity by anion exchange chromatography and size exclusion chromatography as previously described and reported.^30^ Myristoylation of GCAP3 during bacterial expression was accomplished by cotransforming *Escherichia coli* cells with N-myristoyl-transferase from yeast and supplementation with myristic acid, as reported previously. After the hGCAP3 protein was expressed in bacterial cells, the cell pellet was lysed, and inclusion bodies were resuspended in 6M guanidinium hydrochloride for overnight solubilization. The next day, the protein was refolded by dialysis (20 mM Tris-HCl pH 7.5, 2 mM NaCl, 1 mM DTT) and at first purified by anion exchange chromatography with HiTrap Q HP equilibrated in 20 mM Tris-HCl pH 7.5, 2 mM CaCl_2_, and 1 mM DTT, and then, the protein was eluted with a salt gradient from 0.02 to 0.55 M of NaCl. Fractions containing the expressed hGCAP3 protein were further pooled and precipitated by ammonium sulfate and again purified by size exclusion chromatography with Superdex 75 (HiLoad 26/60), which was equilibrated with 20 mM Tris-HCl pH 7.5, 150 mM NaCl, 2 mM CaCl_2_, and 1 mM DTT. SDS-PAGE analyzed the purity of the expressed protein, and samples were exchanged against 50 mM ammonium hydrogen carbonate, lyophilized, and stored at −80 °C for further use.

### Guanylate Cyclase Activity Assay

The double mutant L804P/V902L activity was analyzed by comparing the enzymatic activity with that of V902L. Transiently transfected respective HEK cell pellets were resuspended in 1 mL of 10 mM Hepes/KOH pH 7.4 with 1 mM DTT and a protease inhibitor cocktail. The suspension was incubated for 30 min on ice, followed by cell lysis using a syringe with a 0.7 mm needle. After centrifugation at 13,000 × *g* for 8 min at 4 °C, the cell pellet was resuspended in 100 μL of 50 mM Hepes/KOH pH 7.4, 50 mM KCl, 20 mM NaCl, 1 mM DTT, and a protease inhibitor cocktail. Twenty μL of a GCAP1 solution (10 μM) or water (for the absence of GCAP1) that was previously adjusted to different free Ca^2+^ concentrations using a Ca^2+^/EGTA buffer system exactly as described earlier^31,32^ was used in the assay. For each sample, 10 μL (containing typically 60–100 μg of protein) of respective membrane suspensions were mixed and preincubated for 5 min at room temperature. The reaction started by adding 20 μL of 2.5 × GC buffer (75 mM Mops/KOH pH 7.2, 150 mM KCl, 10 mM NaCl, 2.5 mM DTT, 8.75 mM MgCl_2_, 2.5 mM GTP, 0.75 mM, and 0.4 mM Zaprinast). The reaction mixtures were incubated for 10 min at 30 °C. The reaction was stopped by adding 50 μL of 0.1 M EDTA and incubating at 95 °C for 5 min. Samples were centrifuged for 10 min at 13,000 × *g*. Supernatants were analyzed for the amount of produced cGMP by reversed-phase HPLC using a LiChrospher 100 RP-18 (5 μm) column (Merck, Darmstadt, Germany) exactly as described.^17,29,31^ The detection limit of the assay for cGMP is 2–5 pmol (ref (31) and determination in the Biochemistry group). Measurements were done with three to four biological replicates, each set in technical triplicates, if not stated otherwise, and were evaluated by using SigmaPlot 13.0. Differences in GC activities of biological replicates are due to differences in expression yields of heterologously expressed GC variants (Figures S1–S7 in the Supporting Information).

To check the direct inhibitory effect of Ca^2+^ on GC-E activity, the GC activity assay was performed as mentioned above with the following modification. Half-maximal inhibition of GC-E mutant V902L by Ca^2+^ was determined by setting the free Ca^2+^ concentration to range from 1 to 100 μM without an EGTA-buffer system. All other assay conditions were as described above. Variations of free Mg^2+^ concentration in the assay medium were calculated using the WEBMAXC STANDARD software with proper corrections for pH, salt, and temperature. Incubation was performed in three free Mg^2+^ concentrations of 2, 3.5, and 5 mM.

### Protein Determination, Sodium Dodecyl-Sulfate Polyacrylamide Gel Electrophoresis (SDS-PAGE) and Western Blot Analysis

A modified Bradford assay employing octyl-β-d-glucopyranoside (OGP) to solubilize membrane-bound proteins is used for determination of protein concentration.^33^ Respective membrane suspensions of 5, 10, and 20 μg of total membrane protein containing V902L or L804P were applied to the gel. SDS-PAGE and Western blotting were performed according to established procedures in the laboratory. Primary antibody GC1#3 directed against bovine GC-E^34^ recognized human GC-E and can be used to detect GC-E mutants V902L and V902L/L804P. The dilution of the primary antibody was 1:10,000. Incubation was overnight at 4 °C. A goat antirabbit peroxidase-conjugated antibody (Dianova, Germany) at a concentration of 50% in glycerol was used as a secondary antibody at a dilution of 1:5000. The band intensity was detected and determined using an Azure c400 Gel Imaging System by Azure Biosystems.

### Heterologous Expression of GC-E and Mutants in the HEK293 Cells

Cells were grown on a poly-l-lysine-coated 12 mm coverslip placed in a 24-well plate. After 24 h at a density of 50–70%, they were transiently transfected with respective 0.5 μg of plasmid DNA with 2 μg of PEI. After 48 h of incubation at 37 °C, 5% CO_2_, cells were washed three times (5 min) with PBS (pH 7.4), fixed in 4% paraformaldehyde (PFA) in PBS for 20 min, and then again washed three times (5 min) with PBS (pH 7.4). Then, the cells were incubated with a blocking solution of 5% donkey serum in PBS (pH 7.4) and 0.5% Triton X-100 for 1 h. These were washed one more time (5 min) with PBS (pH 7.4) and then subsequently incubated with the primary antibodies in a similar blocking solution, with primary antibody GC1#3 (1:500), rabbit polyclonal IgG, and anti-calnexin [1:300, calnexin (E-10), sc-46669, mouse monoclonal IgG 2a (Santa Cruz Biotechnology)]. We routinely observed that heterologous expressed GC-E localizes mainly in the ER (colocalization with calnexin), and we measured guanylate cyclase activity in membrane suspensions containing ER.^15^

The next day, cells were again washed three times (5 min) with PBS (pH 7.4) and were further incubated with secondary antibodies [1:200, Alexa Fluor 568 goat antirabbit IgG and 1:200, Alexa Fluor 647 donkey antimouse IgG] for 90 min at room temperature in a blocking solution. The next step was a final washing (three times, 5 min) with PBS (pH 7.4), and then the coverslips were sealed with Mowiol containing DAPI on a microscopic slide and stored at 4 °C until further use. Visualization was done using a fluorescence microscope, Olympus iX2.

### MD Simulation

In the earlier study,^17^ MD simulations were performed on GC-E for a wildtype and a V902L structure.^17^ Employing the same protocol as that in the earlier study, an L804P mutant and a double mutant V902L/L804P were established and simulated. Simulations were based on the structural information provided by Rehkamp et al.^11^ for bovine GC-E. Coordinates of the starting model were provided by Dr. Christian Tüting and Prof. Panagiotis L. Kastritis, both at Martin Luther University Halle-Wittenberg in Germany, upon request. We refer to the human orthologue with a high sequence identity/homology with the bovine variant. The corresponding valine of bovine GC-E is at position 907, and in humans, it is at position 902. Analogously, the double mutant translates to a mutation L809P in bovine GC-E. The connectivity analysis (see below) refers to positions independent of amino acid side chains. Numbering in Figures 4 and 5 relates to the human variant. The simulation was set up through the VIKING online platform^35^ employing the simulation software NAMD.^36,37^ The CHARMM36 force field with CMAP corrections was used.^38−40^

The structure was equilibrated in three phases, gradually lifting harmonic restraints to ensure a stable simulation. For the equilibration protocol, the default parameters of VIKING were chosen. In the first step, 1 ns was simulated with a simulation time step of 1 fs in an NPT (constant number of atoms, constant pressure, and constant temperature) ensemble. Only water and ions were considered free to move. In the second equilibration step, the restraints on the side chains of the protein structure were lifted, and a 2 ns simulation was conducted using the same parameters. In the final equilibration step, all restraints were released, and another 2 ns simulation was run in an NVT (constant number of atoms, constant volume, and constant temperature) ensemble with a pressure of 1.01325 bar.

The production simulation was run for 400 ns in an NVT ensemble with a simulation time step of 2 fs and rigid hydrogen bonds. The other simulation parameters were equal to those from the third equilibration phase. All simulations were performed at a temperature of 310 K.

### Connectivity Analysis

Connectivity networks were constructed based on the final simulation snapshot of all three variants of the GC-E protein structure (wildtype, V902L, L804P, and V902L/L804P). Comparing the so-called amino acid interaction network^41^ between the simulations can be used to identify a change in key residues and potentially suggest potential mutation sites.

The network is constructed from the final simulation snapshot for each GC-E structure variation. Representing the location of each residue by its backbone Cα atom position, the distances between all of the residues were calculated. If the distance between two residues was found below a particular threshold value, the residues were assumed to be connected in the network. Here, a threshold of 8 Å was chosen based on the example of an earlier study.^23^ A binary adjacency matrix can be constructed for each simulation using the threshold. One was employed by Kattnig et al.,^23^ which describes the betweenness of nodes and identifies important hubs in the network (betweenness approach). The second approach was used by Estrada,^22^ which describes the connectivity and the reach of an amino acid residue in the network (reach approach).

### Betweenness Approach

The betweenness is calculated for each residue in each monomer of the dimer such that β_*k*_(*i*) describes the betweenness of residue *i* in monomer *k*. β_*k*_(*i*) is defined as
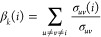
where σ_*uv*_(*i*) is the number of shortest paths connecting nodes *u* and *v*, which pass through the node corresponding to residue *i*, and σ_*uv*_ is the total number of shortest paths connecting *u* and *v*.

Residues with a high betweenness value might be important for pathways through the protein as they are located in strategic intersections throughout the protein, and a mutation in these residues might have a devastating impact on the dynamic properties of the protein structure. As the GC-E structure comprises two monomers and potential experimental validation of proposed mutants would always act on both monomers, the betweenness and the reach values need to be adequately averaged over the individual monomers.

For the network representation, the average betweenness β(*i*) is calculated for the whole dimer from the individual monomer betweenness β_1_(*i*) and β_2_(*i*) for monomers 1 and 2. The final betweenness is then defined as
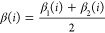


### Reach Approach

The reach approach can be applied to determine the number of other residues that a given residue can reach in *n* steps through the network for every amino acid residue. The more other residues that can be reached with a smaller *n*, the higher the reach of the given amino acid residue becomes. Mathematically speaking, the approach is rooted in the following theorem^42^:

**Theorem 1**. *If A is an adjacency matrix with elements a*_*ij*_*, and A*^*k*^*is the matrix exponential, such that if B* = *A*^2^, *then elements b*_*ij*_*are given by*. *Anelement a*_*jk*_^(*k*)^*in A*^*k*^*is then the number of walks of exact length k from node i to node j in the network described by the adjacency matrix A.*

The theorem can now be used to quantify the connectivity of all of the residues in the protein complex, with weighting factors such that paths to distant residues have less contribution. The matrix exponential function is a perfect fit for the above purpose, given by
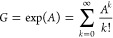


The equation above contains *A*^*k*^ in each summand which yields the adjacency matrices with the number of paths with length *k*, but also a scaling factor 1/*k*!, which devalues longer paths.

The information obtained from *G* can be interpreted in the context of the protein structure as follows. As *a*_*ij*_^(*k*)^ describes the number of paths of lengths *k* from amino acid *i* to amino acid *j* through the connectivity network, a larger value of element *g*_*ij*_ can be interpreted as more options to reach each amino acid *j* from amino acid *i* in shorter trips.

**Figure 6:**
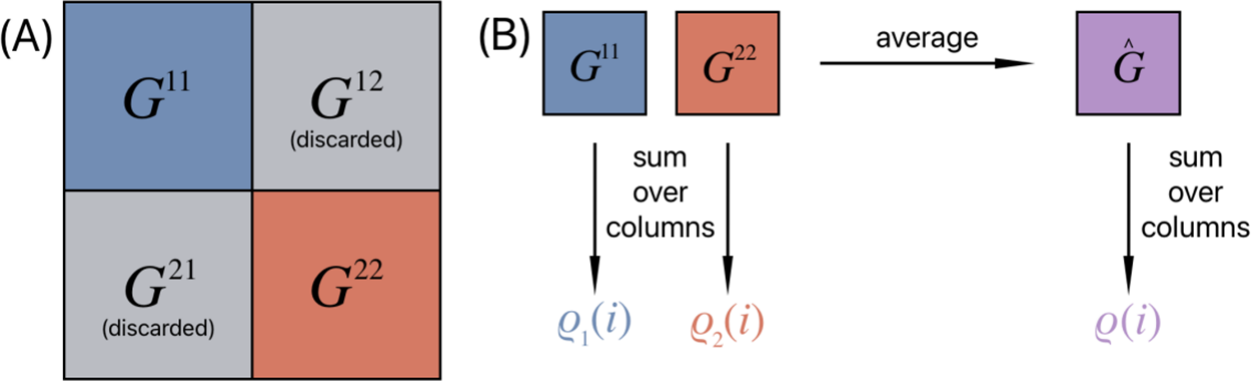
Panel A shows a schematic representation of matrix *G*. Panel B visualizes the averaging process for the individual monomers of GC-E.

The reach value needs to be interpreted for each monomer for the network representation, and an averaging procedure is applied. As each monomer contains 524 amino acid residues (most of the IcD), the total protein contains 1048 residues, and the exponential adjacency matrix *G* defining the network has the size of 1048 × 1048. Subdividing the matrix into four blocks of size 524 × 524 yields two blocks on the diagonal corresponding to the individual monomers denoted by *G*^11^ and *G*^22^. The other two blocks (*G*^12^ and *G*^21^) describing the reach from one monomer to the other are discarded in the analysis. The process is schematically shown in Figure 6. The averaged reach matrix is then the element-wise summation as follows:
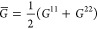


Finally, the sum over column *i* of *G̅* is denoted with ρ(*i*):
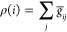
ρ(*i*) is called the reach of residue *i*. A larger value of ρ(*i*) can be interpreted as a greater reach of amino acid *i*, and it is subsequently assumed that the amino acid residue has a greater impact on the overall structure. Conversely, a small reach value would indicate a more isolated residue.

Additionally, to look into residues in their original monomers, ρ_*k*_(*i*) describes the sum over the column of matrices *G*^*kk*^, with the process schematically shown in Figure 6.

### Comparison between WT and V902L Proteins

The reach and distance values are calculated individually for the WT and the V902L mutant. In order to extract the interesting residues from the two structures, the residues with the greatest differences in their values comparing the two versions of the structure are considered.

We, therefore, consider not the absolute values of β(*i*) and ρ(*i*) but their differences between the WT and mutant proteins, defined as
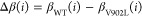
and
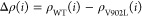


Considering the sign for the individual contributions, positive values in Δβ(*i*) and Δρ(*i*) indicate a greater connectivity in the WT structure compared to the mutant, while a negative value shows a greater connectivity in the mutant compared to the WT. If one now considers the sorted list of residues, the first and last residues are particularly interesting.

### Safety Statement

No unexpected or unusually high safety hazards were encountered.

## Supporting Information Available

Activity profile of GC-E variants (biological replicate); activity profile of GC-E mutant V902L (biological replicate); Ca^2+^-dependent inhibition of the V902L mutant in the absence of GCAP1 or GCAP3 (biological replicates); activity profile of the GC-E variant (biological replicate); expression of V902L and double mutant V902L/L804P in HEK293 cells; and heterologous expression of GC-E and mutants (V902L and V902L/L804P) in HEK293 cells (PDF)

## Accession Codes

GC-E, UniProt: Q02846. GCAP1, NCBI Reference Sequence: NP_001371839.1. GCAP3, NCBI Reference Sequence: NP_005450.3.
