# Supplementary material for: Allosteric Communication of the Dimerization and the Catalytic Domain in Photoreceptor Guanylate Cyclase
Source: Biochemistry. 2024 Aug 23;63(17):2131–40. doi: 10.1021/acs.biochem.4c00170 (PMC11375764; doi:10.1021/acs.biochem.4c00170)
Supplement: Supplementary file 1 — bi4c00170_si_001.pdf [file bi4c00170_si_001.pdf]

# Supporting information

# Allosteric communication of the dimerization and the catalytic domain in photoreceptor guanylate cyclase

Manisha Kumari Shahu[1], Fabian Schuhmann[2,3], Siu Ying Wong[3], Ilia A. Solov'yov[3,4,5] and Karl-Wilhelm Koch[1,4]

[1]Carl von Ossietzky Universität Oldenburg, Department of Neuroscience, Carl-von-Ossietzky-Str. 9-11, Oldenburg, 26129, Germany.

[2]Niels Bohr International Academy, Niels Bohr Institute, University of Copenhagen, Blegdamsvej 17, 2100 Copenhagen, Denmark

[3]Carl von Ossietzky Universität Oldenburg, Institute of Physics, Carl-von-Ossietzky-Str. 9-11, Oldenburg, 26129, Germany.

[4]Carl von Ossietzky Universität Oldenburg, Research Centre for Neurosensory Science, Carl-von-Ossietzky-Str. 9-11, Oldenburg, 26129, Germany.

[5]Center for Nanoscale Dynamics (CENAD), Carl von Ossietzky Universität Oldenburg, Institute of Physics, Ammerländer Heerstr. 114-118, 26129 Oldenburg, Germany.

Corresponding authors: karl.w.koch@uol.de

**Figure S1**

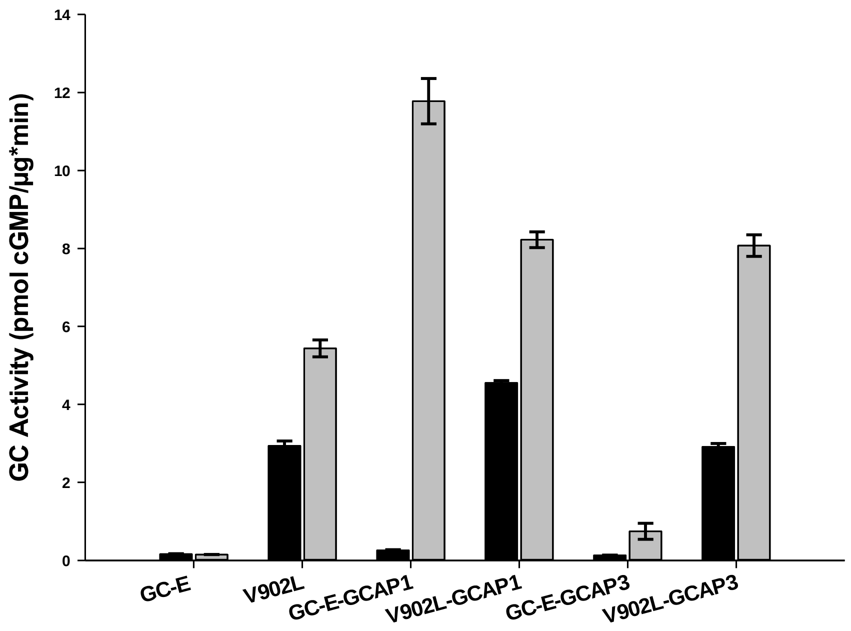


**Figure S1. Activity profile of GC-E variants.** WT and mutant V902L were activated with GCAP1 or GCAP3 at high (33 µM, black bars) or low (<10 nM, gray bars) free [$Ca^{2+}$]. The x-fold activation of GC-E WT with GCAP1 is 45.3 and with GCAP3 is 5.8. The x-fold activation of V902L without GCAPs is 1.9; in the presence of GCAP1 is 1.8 and with GCAP3 it is 2.8. Error bars are s.d of technical replicates.

**Figure S2**

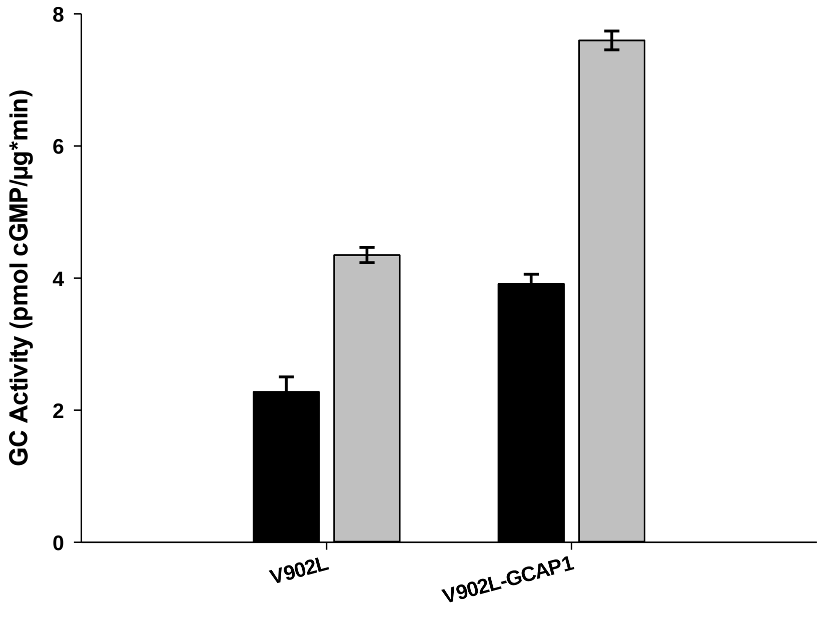


**Figure S2. Activity profile of GC-E mutant V902L**. Assay was done in the absence and presence of GCAP1 at high (33 µM, black bars) or low (<10 nM, gray bars) free [$Ca^{2+}$]. The

x-fold activation of V902L without GCAP1 is 1.9; in the presence of GCAP1 is 1.9. Error bars are s.d of technical replicates.

**Figure S3**

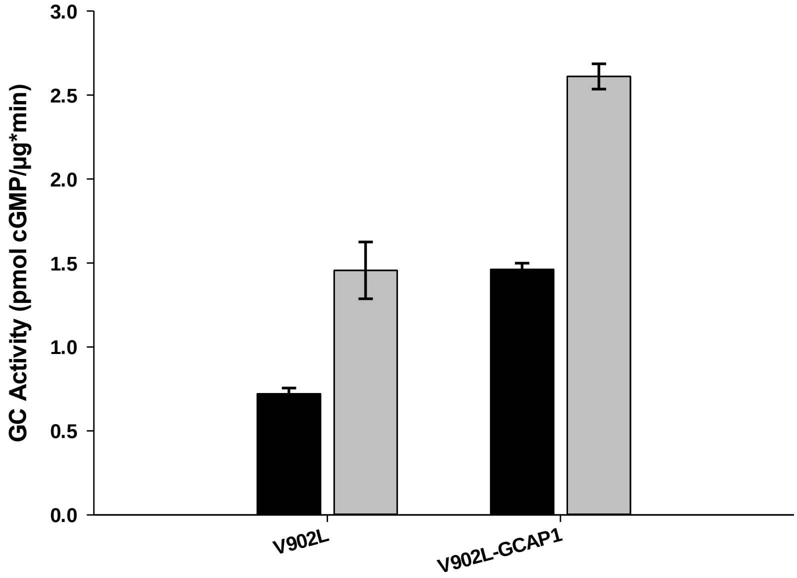


**Figure S3. Activity profile of GC-E mutant V902L.** Assay was done in the absence and presence of GCAP1 at high (33 µM, black bars) or low (<10 nM, gray bars) free [$Ca^{2+}$]. The x-fold activation of V902L without GCAP1 is 2; in the presence of GCAP1 is 1.8. Error bars are s.d of technical replicates.

**Figure S4**

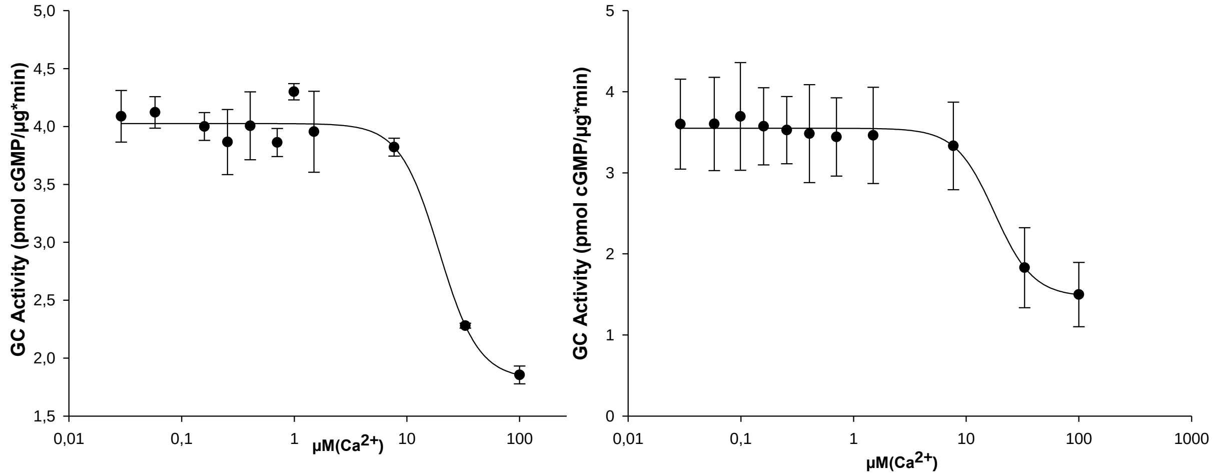


**Figure S4. $Ca^{2+}$-dependent inhibition of the V902L mutant in the absence of GCAP1 or GCAP3.** Free [$Ca^{2+}$] was varied as indicated and half-maximal inhibition was at 19.3 µM free [$Ca^{2+}$] (left graph) and 17.8 µM [$Ca^{2+}$] (right graph), (biological replicates, error bars are s.d.).

**Figure S5**

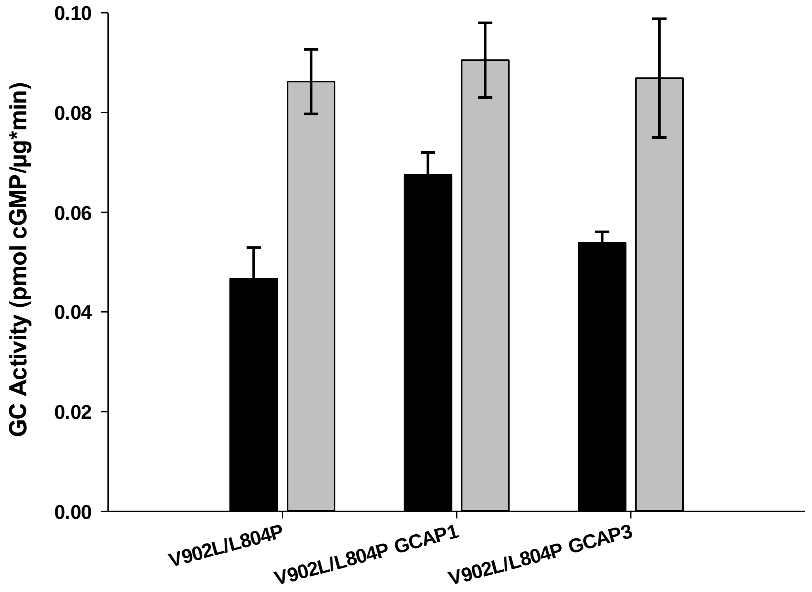


**Figure S5. Activity profile of GC-E variants.** Double mutant V902L/L804P was activated with GCAP1 or GCAP3 at high (33 µM, black bars) or low (<10 nM, gray bars) free [$Ca^{2+}$]. The x-fold activation of double mutant without GCAP1 is 1.8; in the presence of GCAP1 it is 1.3 and with GCAP3 it is 1.6. Error bars are s.d of technical replicates.

**Figure S6**

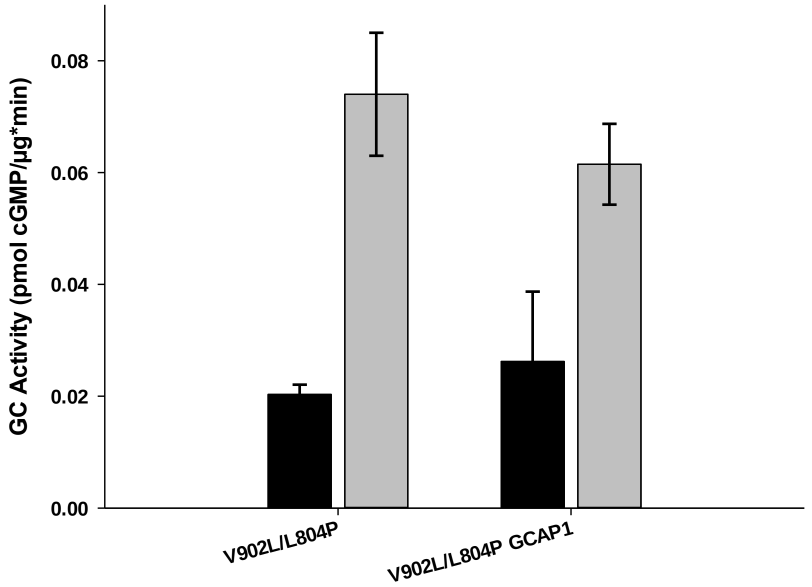


**Figure S6. Activity profile of GC-E variant.** Double mutant V902L/L804P was activated with GCAP1 at high (33 µM, black bars) or low (<10 nM, gray bars) free [$Ca^{2+}$]. The x-fold activation of double mutant without GCAP1 is 3.6 and is 2.3 in the presence of GCAP1. Error bars are s.d of technical replicates.

**Figure S7**

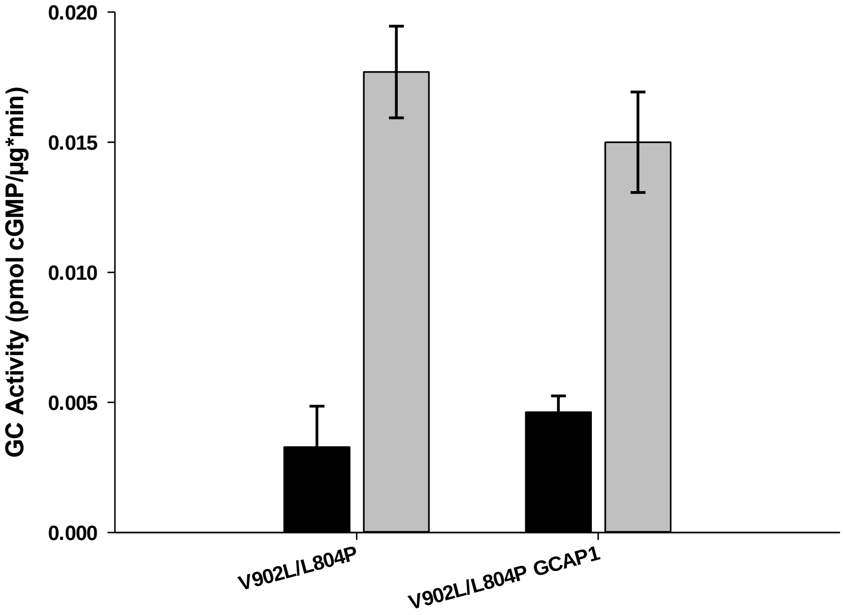


**Figure S7. Activity profile of GC-E variant.** Double mutant V902L/L804P was activated with GCAP1 at high (33 µM, black bars) or low (<10 nM, gray bars) free [$Ca^{2+}$]. The x-fold activation of double mutant without GCAP1 is 5.4 and is 3 in the presence of GCAP1. Error bars are s.d of technical replicates.

**Figure S8**

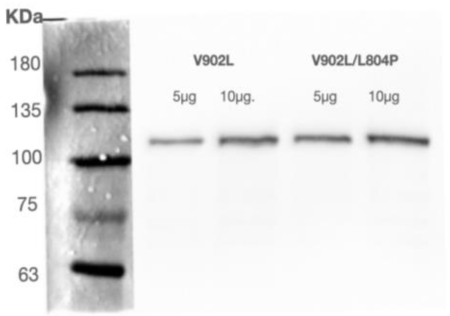


**Figure S8. Expression of V902L and double mutant V902L/L804P in HEK293 cells.** Expression level of mutants was compared in HEK293 cell membrane for its expression profile using immunoblotting. Five µg and ten µg of total protein amount for each membrane sample was analyzed by SDS-polyacrylamide gel electrophoresis. The samples were detected by the specific antibody anti-GC1#3 and ECL imaging (see Methods part in main text).

**Figure S9.**

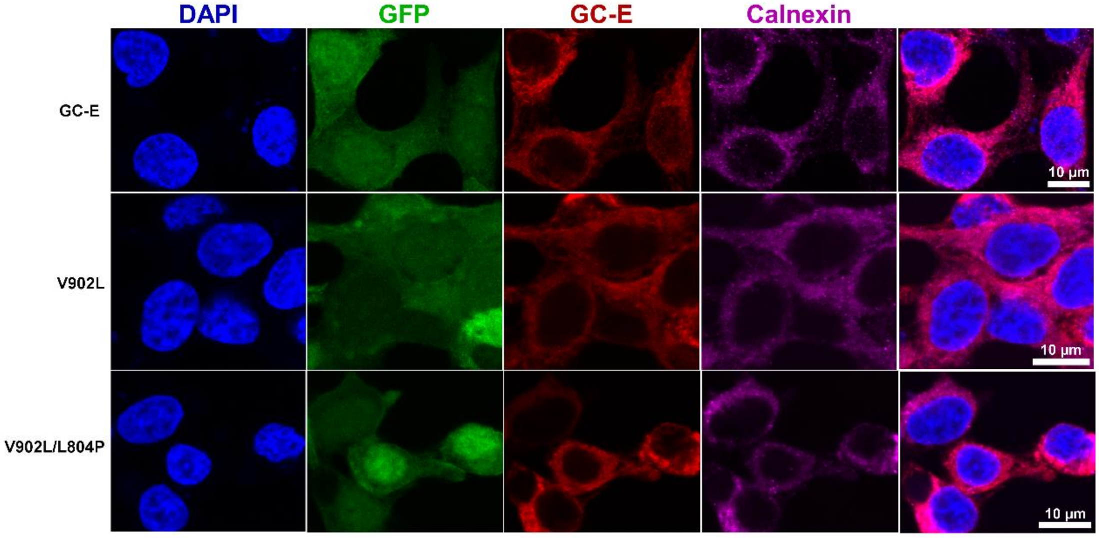


**Figure S9. Heterologous expression of GC-E and mutants (V902L, V902L/L804P) in HEK293 cells**. Confocal images of transfected cells probed with different antibodies. Transfected cells are visualized with GFP (green) and DAPI staining (nucleus), GC-E variants are recognized by an anti-GC-E specific antibody (#3, red), Endoplasmic reticulum (ER) is stained with the ER marker Calnexin (magenta). No difference has been observed in the localization of the mutants compared to the WT GC-E. An overlay is seen on the most right panels. Scale bar is 10 µm.
